# Transcriptome Remodeling in *Arabidopsis*: A Response to Heterologous Poplar MSL-lncRNAs Overexpression

**DOI:** 10.3390/plants13202906

**Published:** 2024-10-17

**Authors:** Jinyan Mao, Qianhua Tang, Huaitong Wu, Yingnan Chen

**Affiliations:** State Key Laboratory of Tree Genetics and Breeding, Co-Innovation Center for Sustainable Forestry in Southern China, Key Laboratory of Forest Genetics & Biotechnology of Ministry of Education, Nanjing Forestry University, Nanjing 210037, China; maojinyan@njfu.edu.cn (J.M.); tangqianhua@njfu.edu.cn (Q.T.); wuht@njfu.edu.cn (H.W.)

**Keywords:** MSL-lncRNAs, overexpression, transcriptome analysis, molecular regulatory

## Abstract

Stamens are vital reproductive organs in angiosperms, essential for plant growth, reproduction, and development. The genetic regulation and molecular mechanisms underlying stamen development are, however, complex and varied among different plant species. MSL-lncRNAs, a gene specific to the Y chromosome of *Populus deltoides*, is predominantly expressed in male flower buds. Heterologous expression of MSL-lncRNAs in *Arabidopsis thaliana* resulted in an increase in both stamen and anther count, without affecting pistil development or seed set. To reveal the molecular regulatory network influenced by MSL-lncRNAs on stamen development, we conducted transcriptome sequencing of flowers from both wild-type and MSL-lncRNAs-overexpressing *Arabidopsis*. A total of 678 differentially expressed genes were identified between wild-type and transgenic *Arabidopsis*. Among these, 20 were classified as transcription factors, suggesting a role for these regulatory proteins in stamen development. GO enrichment analysis revealed that the differentially expressed genes were significantly associated with processes such as pollen formation, polysaccharide catabolic processes, and secondary metabolism. KEGG pathway analysis indicated that MSL-lncRNAs might promote stamen development by upregulating genes involved in the phenylpropanoid biosynthesis pathway. The top three upregulated genes, all featuring the DUF295 domain, were found to harbor an F-box motif at their N-termini, which is implicated in stamen development. Additionally, in transgenic *Arabidopsis* flowers, genes implicated in tapetum formation and anther development were also observed to be upregulated, implying a potential role for MSL-lncRNAs in modulating pollen development through the positive regulation of these genes. The findings from this study establish a theoretical framework for elucidating the genetic control exerted by MSL-lncRNAs over stamen and pollen development.

## 1. Introduction

Flowering is a critical phase in plant growth and development, serving as a vital means of reproduction. However, the flowering process in plants is a highly complex process, regulated by numerous genes and affected by various external factors [1]. Early flower development typically involves three stages: floral determination, floral initiation, and floral organ formation [2]. Upon receiving signals indicating self-flowering, the vegetative meristem transforms into the inflorescence meristem, initiating the flowering process influenced by these external cues. This meristem then progresses to the floral meristem, gradually giving rise to floral primordia, which subsequently develop into the organs. These floral primordia then develop sequentially, progressively forming the floral organs.

In dicotyledonous plants, flowers generally consist of four primary parts, arranged from the outermost to the innermost as sepals, petals, stamens, and pistils [3]. *Arabidopsis thaliana*, a model organism for dicots, exemplifies the typical structural features of dicot flowers. The development of *Arabidopsis* flowers is divided into 20 distinct stages, from the formation of flower primordia to seed maturation [4]. Meyerowitz and Coen established the classic ABC model of flower organ development based on studies of *Arabidopsis* and *Antirrhinum majus*, which involves three classes of genes regulating development [5,6]. Further research across various species has refined this model into the ABCDE model, which includes five gene classes [7,8]. All identified genes, except the *AP2* gene from the AP2/ERF family, belong to the MADS-box gene family. Additionally, transcription factors like MYB, WRKY, bHLH, bZIP, NAC, ERF, and CO play crucial roles in regulating floral organ development at different stages [9,10,11,12,13,14,15].

The pistil and stamen are the reproductive organs of angiosperms. The stamen, comprising anthers and filaments, serves as the site for male gametes, with filaments providing structural support and nutrition to the anthers. Within the anther, male sporogenous cells differentiate and undergo meiosis to produce microspores, which develop into pollen grains. Other cell types contribute to pollen maturation, protection, or release [16]. Once mature pollen grains are released from the anthers, they adhere to the stigma and undergo a series of processes, including germination, pollen tube growth, and sperm cell transport to the embryo sac. This culminates in zygote formation, completing the double fertilization process in plants [17,18]. Previous studies have shown that certain transcription factors can influence stamen development. Genes within the *AGAMOUS* evolutionary branch of the MADS-box family are responsible for determining the characteristics of stamens and carpels [19]. Members of the MIKC group are involved in pollen maturation in both *Arabidopsis* and rice [20]. For example, *GhMYB24*, a MYB transcription factor in cotton, is expressed in pollen; its overexpression in *Arabidopsis* inhibits filament elongation and anther dehiscence and reduces pollen viability, dependent on the phenylpropanoid biosynthetic pathway. Additionally, genes associated with jasmonic acid biosynthesis and signaling are upregulated in transgenic plants [21]. Another transcription factor, *GhWRKY22*, has been identified in cotton and is shown to induce male fertility defects in transgenic *Arabidopsis*. Molecular interactions suggest that *GhWRKY22* regulates anther and pollen development by binding to the promoters of jasmonate repressor genes such as *JAZ1* and *JAZ8* [22].

Poplars are widely distributed in the Northern Hemisphere, with approximately 30 species. They exhibit characteristics such as rapid growth and strong stress resistance [23,24]. From the perspective of individual gender, almost all poplar trees are dioecious. Previous research by our laboratory has demonstrated that *Populus deltoides* is a dioecious species, with *MSL* genes and *FERR-R* genes present in specific regions of the Y chromosome [25]. Expression analysis of flower buds at different stages revealed that the *MSL* gene is expressed in male flower buds of *P. deltoides* but not in female flower buds. Transcriptome sequencing and strand-specific lncRNA sequencing of male flower buds indicated that *MSL* functions by producing lncRNA. The *MSL* gene features a high degree of repetitive sequences and belongs to the Gypsy family of LTR retrotransposons. We constructed an overexpression vector and performed heterologous transformation of *Arabidopsis*, discovering that the *MSL* gene enhances stamen development without affecting pistil development [25]. Additionally, based on sequence alignment, we segmented the *MSL* gene into three partial sequences and constructed three overexpression vectors for subsequent transformation experiments in *Arabidopsis thaliana*. These findings indicate that the partial sequences of the *MSL* gene also affect stamen development without influencing pistil development [26]. However, the specific roles and pathways of MSL-lncRNAs in promoting stamen development remain unclear. In this study, we conducted transcriptome sequencing of transgenic *Arabidopsis* flowers to identify differentially expressed genes (DEGs) and performed functional enrichment analysis. Our aim was to elucidate the molecular regulatory network of MSL-lncRNAs, providing a theoretical foundation for exploring their interaction mechanism.

## 2. Results

### 2.1. RNA Sequencing and Alignment to the Arabidopsis Genome

**Figure 1:**
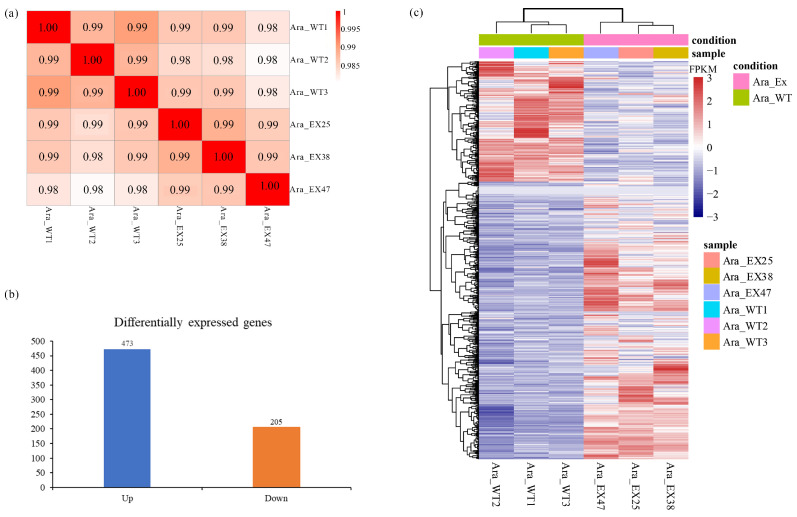
Transcriptome data analysis. (**a**) Correlation analysis among six samples. (**b**) Bar Chart of the number of differentially expressed genes. (**c**) Cluster analysis of DEGs collected in six samples. The normalized FPKM expression is indicated by the row Z-score, where red represents upregulated genes and blue represents downregulated genes in every sample.

Six cDNA libraries were constructed from high-quality RNA and sequenced using the Illumina NovaSeq 6000 platform. The libraries generated a total of 44,288,032; 38,908,522; 45,654,690; 40,657,346; 4,114,460; and 40,870,856 clean reads, respectively. With the overexpression line Ara_EX47, the mapping rate of reads to the genome for the remaining five libraries exceeded 97% (Table 1). All samples demonstrated high correlation, clustering together with Pearson correlation coefficients ranging from 0.985 to 0.995 (Figure 1a). These results indicate both high sequencing quality and robust experimental reliability.

### 2.2. Analysis of DEGs Between Wild-Type and Transgenic Arabidopsis

The gene expression profiles of wild-type and transgenic *Arabidopsis* were analyzed and compared to identify DEGs. Applying thresholds of logFC ≥ 1 or logFC ≤ 1 and a *p*-value < 0.05, a total of 678 DEGs were identified (Supplementary Table S1), comprising 473 upregulated and 205 downregulated genes (Figure 1b). Hierarchical cluster analysis of DEGs demonstrated that the expression patterns of related genes could be distinctly differentiated between three wild-type and three transgenic samples (Figure 1c). These findings indicate that the identified DEGs effectively reflect the impact of MSL-lnRNAs overexpression.

**Figure 2:**
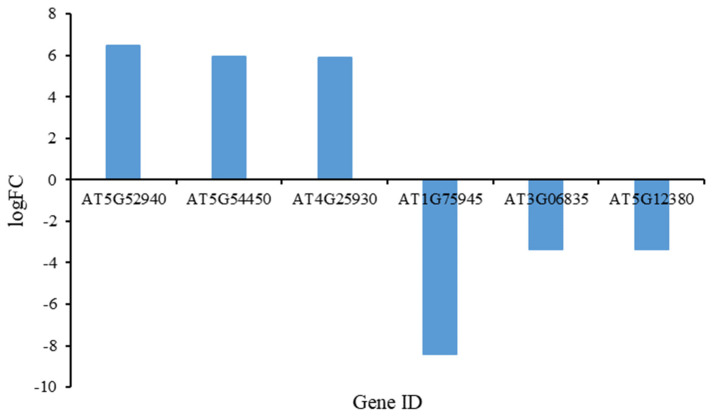
Bar chart displaying the top three upregulated and bottom three downregulated genes based on log-fold change (logFC) values.

**Figure 3:**
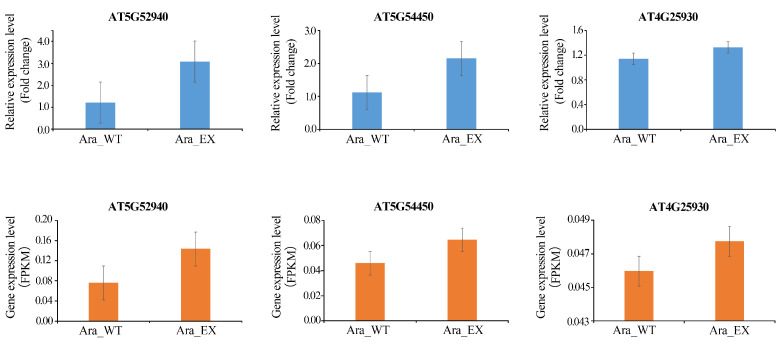
Validation of RNA-seq results using qRT-PCR analysis. The top three histograms depict the relative expression levels from qRT-PCR, with fold change values shown as the mean ± standard deviation across three independent experiments. The bottom three histograms illustrate the FPKM values derived from RNA-seq data.

Among the DEGs, we selected the top three with the highest and the bottom three with the lowest fold change (FC) values for analysis (Figure 2). The FPKM values for these genes can be found in Supplementary Table S2. A search of the National Center for Biotechnology Information (NCBI) database revealed that AT5G52940 (Chr5: 21472103-21473602), AT5G54450 (Chr5: 22108273-22109379), and AT4G25930 (Chr4: 13168127-13169393) are classified as hypothetical proteins (DUF295) containing the domain of Unknown Function 295. The C-terminal DUF295 domain is often accompanied by an N-terminal F-box domain [27], and studies indicate that F-box family members are involved in flower development [28]. Additionally, AT1G75945 (Chr1: 28515245-28515916) is characterized as an uncharacterized protein, while AT3G06835 (Chr3: 16071671-16072151) is annotated as an lncRNA, with its function yet to be reported. AT5G12380 (Chr5: 4009168-4010687) belongs to the Annexin family and may be associated with abiotic stress responses in plants. These findings indicate that the three upregulated genes may be linked to stamen development and could play a role in this process. To verify the reliability of the RNA-seq results, we performed qRT-PCR validation on these three genes. The results showed that the expression patterns of these genes were consistent with those obtained from transcriptome sequencing (Figure 3), further corroborating the reliability of the RNA-seq data.

### 2.3. Expression Profiles of Transcription Factor Genes During Flower Development

**Figure 4:**
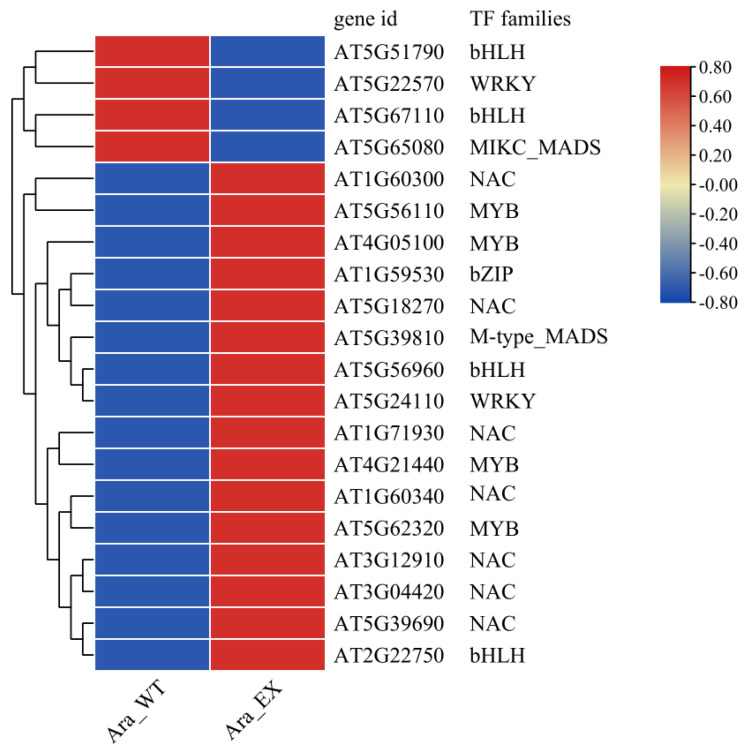
Heatmap of differentially expressed transcription factors based on FPKM values. Normalized transcription factor expression is indicated by the row Z-score where red represents upregulated genes and blue represents downregulated genes.

Transcription factors (TFs) play a crucial role in flower development. The MADs-box, MYB, bHLH, bZIP, NAC, and WRKY families are among the most prominent TF families involved in this process [29,30,31,32,33]. In this study, we identified a total of 20 differentially expressed TFs belonging to these six TF families (Figure 4). The downregulated MADs-box gene, AT5G65080 (*MAF5*), was a homolog of the flowering repressor FLOWERING LOCUS C (*FLC*). The upregulated MYB gene, AT5G62320 (*MYB99*), has been reported to influence pollen wall formation and pollen viability [34]. Three upregulated NAC members (AT1G60300, AT1G71930, and AT5G18270), which may be associated with anther dehiscence, were also identified. The remaining differentially expressed MAD-box, MYB, bHLH, bZIP, NAC, and WRKY genes—specifically, One MAD-box, three MYB, four bHLH, one bZIP, four NAC, and two WRKY—may also play potential roles in flower development, but further verification is needed.

### 2.4. Annotation and Functional Classification of the Transcriptome

**Figure 5:**
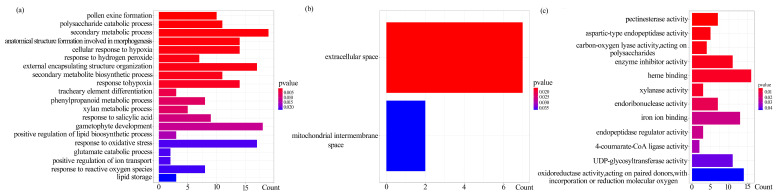
GO enrichment analysis of DEGs. (**a**) Biological process enrichment analysis. (**b**) Cellular component enrichment analysis. (**c**) Molecular function enrichment analysis.

An analysis of 678 DEGs was performed to identify major functional categories during the later stages of flower development in overexpressed MSL-lncRNAs. Several significantly enriched Gene Ontology (GO) terms were associated with pollen exine formation, secondary metabolic processes, external encapsulating structure organization, and gametophyte development. A total of 408 genes were classified into 58 GO functional groups within three main GO categories (Supplementary Table S3). The most prevalent GO terms in the biological process category include metabolic process, multicellular organismal process, developmental process, cellular process, and response to stimulus. Figure 5a illustrates the top 20 GO functional groups in this category. Specifically, ten genes were annotated with GO terms related to pollen exine formation (GO:0010584), 19 genes with secondary metabolic process (GO:0019748), 14 genes were associated with anatomical structure formation involved in morphogenesis (GO:0048646), and 18 genes were linked to gametophyte development (GO:0048229) (Figure 5a, Supplementary Figure S1). The most significantly enriched GO terms pertained to the extracellular space (GO:0005615) and mitochondrial intermembrane space (GO:0005758) (Figure 5b, Supplementary Figure S2). In the molecular function category, GO terms related to pectinesterase activity (GO:0030599), enzyme inhibitor activity (GO:0004857), and heme binding (GO:0020037) were identified (Figure 5c, Supplementary Figure S3).

Previous studies have reported that *SHT* genes are specifically expressed in anther chorioallantoic cells [35], while *CYP7041* and *CYP703* (*CYP703A2*) are expressed in developing anthers [36,37]. The *QRT3* gene is implicated in the degradation of the pollen mother cell wall during microspore development [38]. In our study, *SHT* and *CYP703* were found to be involved in pollen exine formation, anatomical structure formation involved in morphogenesis, external encapsulating structure organization, and gametophyte development. Notably, *SHT*, *CYP703*, *CYP7041,* and *QRT3* were all linked to pollen exine formation.

**Figure 6:**
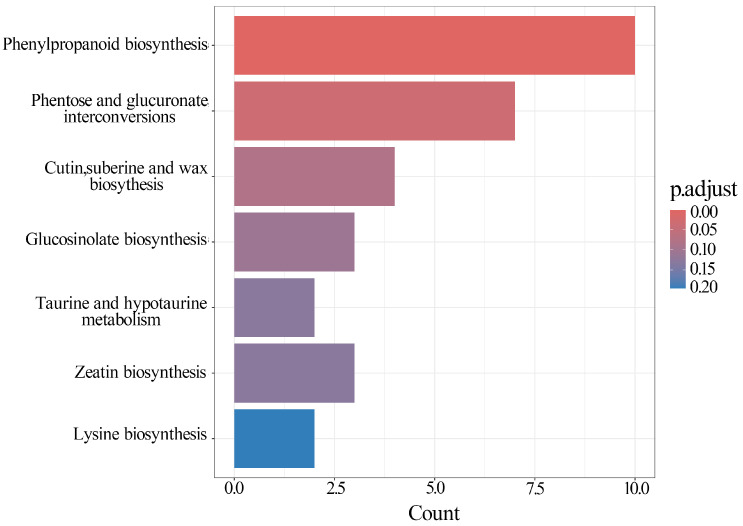
KEGG enrichment analysis of DEGs. The *X*-axis represents the number of DEGs enriched in specific metabolic pathways. The color gradient from red to blue denotes adjusted *p*-values: red for the smallest (0.00), purple for moderate (0.10), and blue for the largest (0.20).

In addition, Kyoto Encyclopedia of Genes and Genomes (KEGG) enrichment analysis (*p*. adjust < 0.5) identified 31 DEGs mapped to 7 KEGG pathways (Figure 6, Supplementary Table S4), including phenylpropanoid biosynthesis; pentose and glucuronate interconversions; cutin, suberine, and wax biosynthesis; and glucosinolate biosynthesis, among others. However, among these pathways, only phenylpropanoid biosynthesis (*p*. adjust < 0.05) and pentose and glucuronate interconversions (*p*. adjust < 0.05) were significantly enriched (Figure 6, Supplementary Table S4).

### 2.5. KEGG-Annotated Genes Involved in Phenylpropanoid Biosynthesis Pathway

**Figure 7:**
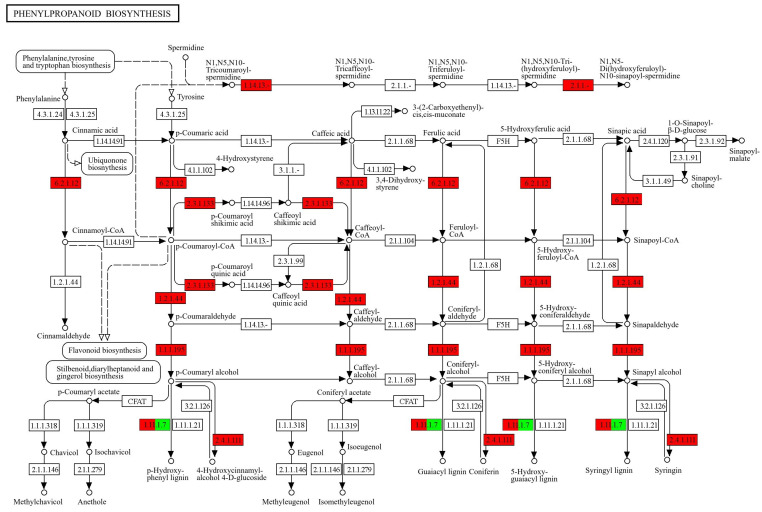
Differential expression levels of genes related to phenylpropanoid biosynthesis identified by KEGG annotation. The enzymes marked with the red boxes are associated with the upregulation of proteins, while those marked with the green boxes are associated with the downregulation of proteins.

According to the results of the KEGG enrichment analysis, the phenylpropanoid biosynthesis pathway (map 00940) was the most significantly enriched pathway, with a total of 10 genes identified in this pathway. The AT2G19070 gene is assigned with shikimate O-hydroxycinnamoyltransferase [EC:2.3.1.133] and the AT1G67990 gene is linked to putative caffeoyl-CoA 3-O-methyltransferase [EC:2.1.1.-]. The AT5G66690 gene corresponds to coniferyl-alcohol glucosyltransferase [EC:2.4.1.111] and the AT1G74550 gene is classified as cytochrome P450 family 98 subfamily A8 [EC:1.14.13.-]. Additionally, the AT1G62940 gene is linked to 4-coumarate-CoA ligase [EC:6.2.1.12], AT1G80820 gene to cinnamoyl-CoA reductase [EC:1.2.1.44], and AT2G34790 gene to cinnamyl-alcohol dehydrogenase [EC:1.1.1.195]. Furthermore, the AT5G51890, AT5G15180, and AT5G19880 genes are assigned to peroxidase [EC:1.11.1.7] (Figure 7). Notably, the AT5G51890 and AT5G15180 genes were upregulated, while the AT5G19880 gene showed downregulation.

**Figure 8:**
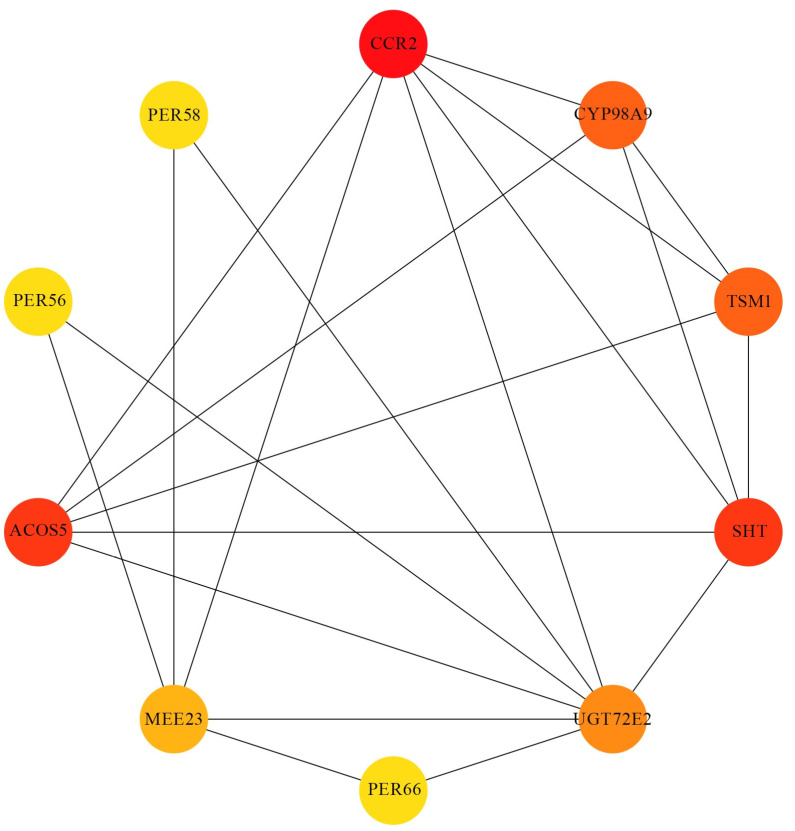
Protein–protein interaction network in *Arabidopsis*. Each node represents a protein, with the protein name displayed inside. Arcs denote interactions between proteins, and color coding reflects interaction strength: red for high, orange for moderate, and yellow for low interaction degrees.

We analyzed some genes involved in the phenylpropanoid biosynthesis pathway, including 10 genes: *SHT* (AT2G19070), *TSM1* (AT1G67990), *UGT72E2* (AT5G66690), *CYP98A9* (AT1G74550), *CCR2* (AT1G80820), *ACOS5* (AT1G62940), *MEE23* (AT2G34790), *PER66* (AT5G51890), *PER56* (AT5G15180), and *PER58* (AT5G19880). Among these genes, AT5G19880 is the only one whose expression level was downregulated, while the expression levels of the other genes were all upregulated. Furthermore, we conducted a separate analysis of the protein–protein interactions among the 10 differentially expressed genes involved in phenylpropanoid biosynthesis using the STRING database. We then constructed a protein–protein interaction network, which is presented in Figure 8. The results indicate that CCR2, SHT, and ACOS5 have a high number of connections in the protein–protein interaction network, suggesting that they may be key regulatory factors with significant roles in stamen development.

## 3. Discussion

The stamen is a vital reproductive structure in plants, consisting of two primary elements: the filament and the anther. The anther plays a crucial role in pollen production. The pollen wall protects the sperm with a dual-layer structure composed of an outer wall and an inner wall. In the model plant *Arabidopsis*, the floral architecture features a unique characteristic known as tetradynamous, which is defined by the presence of four elongated stamens accompanied by two shorter ones; the former developing slightly earlier than the latter [39].

Previous research has demonstrated the intriguing effects of MSL-lncRNAs overexpression, resulting in a notable alteration in the number of stamens in *Arabidopsis*. This was evident in the emergence of six elongated stamens, as well as an unexpected seven or eight stamens. Additionally, the MSL-lncRNAs gene and its homologous sequences are present in the poplar genome but absent in the *Arabidopsis* genome. Consequently, utilizing RNA sequencing to accurately delineate the alterations in gene expression profiles between wild-type and transgenic *Arabidopsis* flowers is crucial for studying the molecular regulatory network of MSL-lncRNAs. Through transcriptomic comparisons, our study successfully identified 678 DEGs. The ABC model of flower development proposes that stamen development is determined by both Class B and Class C genes [6]. Class B genes affect only the stamens, not the pistils. The Study revealed that the F-box *UFO* gene interacts with *ASK1* and *LEAFY*, leading to the upregulation of Class B gene expression [40]. In our results, AT5G52940, AT5G54450, and AT4G25930 possess the F-box domain, suggesting that they may be potential genes involved in the co-regulation of stamen development by MSL-lncRNAs.

Transcription factors represent a significant proportion of eukaryotic genomes, with many categorized into distinct gene families based on their DNA-binding domains [41]. Notably, several transcription factors are crucial for flower development, including stamen development. A total of 58 transcription factor families have been identified in plant genomes [42]. A comprehensive analysis of 10 transcription factor families associated with flower development in *Arabidopsis thaliana* revealed a marked absence of members from the TCP, CO-like, LFY, and AP2 families among 678 DEGs. In contrast, the MADS-box, MYB, bHLH, and NAC families were prominently represented, with a total of 20 DEGs identified, 16 of which were upregulated. The *MAF5* gene, a member of the MADS-box transcription factor family featuring a K-box region, is a homolog of the flowering repressor gene *FLC* in *Arabidopsis* [43]. In our transgenic plants, *MAF5* expression was downregulated. Within the MYB transcription factor family, research has shown that *MYB80* is essential for tapetum and pollen development [44], while *MYB99* plays a role in pollen wall formation and survival. Furthermore, *MYB99* interacts with *MYB21* and *MYB24*, linking to the phenylpropanoid pathway [34]]. *MYB74* and MYB102, identified as negative regulators of plant growth, are implicated in growth control and abiotic stress responses [45]. The four MYB genes examined in our study, AT4G05100 (*MYB74*), AT4G21440 (*MYB102*), AT5G56110 (*MYB80*), and AT5G62320 (*MYB99*), were all upregulated in transgenic *Arabidopsis*. The secondary cell wall is primarily located in the xylem, fibers, and anther cells [46]. Research indicates that *Arabidopsis* NAC transcription factors *NST1* and *NST2* are key regulators of secondary wall thickening, essential for anther dehiscence [47]. Among the eight identified NAC family members, AT1G60300, AT1G71930, and AT5G18270 are involved in synthesizing the secondary cell wall, suggesting their potential role in *Arabidopsis* anther dehiscence.

The overexpression of MSL-lncRNAs in *Arabidopsis* leads to an increase in the number of stamens and anthers, indicating significant changes in the expression of genes related to stamen development. In *Arabidopsis*, *CYP703A2* plays a crucial role in the biosynthesis of precursors necessary for the formation of the anther cuticle and pollen exine [37]. *ACOS5* and *PKSA* are located in the endoplasmic reticulum of the tapetum layer [48]. Additionally, *PKSB* and *TKPR1* interact to form a multi-enzyme complex, with *ACOS5* also interacting with *CYP703A2* [48]. Studies on *OsTKPR1* in rice suggest that its functional deficiency can delay the degradation of the tapetum layer, thereby impairing the formation of the pollen outer wall [49]. In transgenic *Arabidopsis*, the upregulation of *CYP703A2*, *ACOS5*, *PKSA*, and *MYB80* genes (Supplementary Table S1) suggests that these genes may be co-expressed with MSL-lncRNAs, collectively regulating stamen development. GO analysis indicates that the *ACOS5* and *CYP703A2* genes are involved in pollen exine formation.

Jasmonic acid (JA) and gibberellic acid (GA) are essential hormones for plant growth and development, and they have been demonstrated to regulate pathways related to anther and pollen development [21,50]. Notably, the overexpression of cotton *GhMYB24* in *Arabidopsis* affects stamen development, implicating the synthesis of JA-related genes and the phenylpropanoid biosynthetic pathway [21]. In our research, while differentially expressed genes related to the anther/pollen pathway were not enriched in the JA and GA pathways, several functions associated with pollen development were significantly enriched. These include pollen exine formation, anatomical structure formation involved in morphogenesis, external encapsulating structure organization, and gametophyte development. Our KEGG enrichment analysis indicated that the phenylpropanoid biosynthetic pathway exhibited the highest abundance of DEGs, suggesting that MSL-lncRNAs may influence stamen and pollen development by participating in this pathway.

## 4. Materials and Methods

### 4.1. Plant Materials and Growth Conditions

A. thaliana ecotype Columbia-0 (Col-0) was used as the wild-type (WT). Seeds of three independent MSL-lncRNAs overexpression transgenic lines (Ara_EX25, Ara_EX38, and Ara_EX47) were obtained from Xue et al. [25]. Both WT and transgenic *Arabidopsis* seeds underwent surface sterilization with a 70% ethanol solution for 30 s, followed by treatment with a 4% sodium hypochlorite solution for 10 min. Subsequently, the seeds were germinated on agar plates containing half-strength Murashige and Skoog media (1% *w*/*v*) supplemented with 1% sucrose (pH 5.8). To ensure synchronized germination, the *Arabidopsis* seeds were subjected to vernalization at 4 °C for 3 days. Fourteen days post-germination, all seedlings were transferred to soil and cultivated at 22 °C under a photoperiod of 16 h light and 8 h darkness, with the environmental humidity maintained between 40% and 50%. Flowers were collected and immediately submerged in liquid nitrogen for transcriptome sequencing.

### 4.2. RNA Sequencing Data and Differentially Expressed Gene Analysis

Total RNA was extracted from *Arabidopsis* flowers using the RNAprep Pure Plant Kit (Tiangen Co., Ltd., Nanjing, China). The purity, concentration, and integrity of the RNA samples were assessed, followed by mRNA enrichment and ribosomal RNA subtraction. cDNA reverse transcription was then performed, and a sequencing library with adapters was prepared. Six cDNA libraries were generated for quality verification. High-throughput sequencing was conducted using the Illumina NovaSeq 6000 (Illumina, CA, USA) platform, with a read length of PE150. Clean reads were obtained by removing adapter-contaminated reads, low-quality reads, and those containing more than 5% ambiguous bases (N). These clean reads were mapped to the *Arabidopsis* genome (TAIR 11 [51]) using HISAT 2.2.1 software [52]. Gene expression levels were estimated using FPKM (Fragments Per Kilobase of transcript per Million mapped reads) [53], and Pearson’s correlation coefficients were calculated to analyze sample correlations. Differential gene expression between wild-type and transgenic *Arabidopsis* was assessed using the DESeq2 package in R [54]. Genes with log(Fold Change) ≥ 1 or log(Fold Change) ≤ 1 and a *p*-value < 0.05 were considered significantly differentially expressed. The number of upregulated and downregulated genes was subsequently calculated. 

The *Arabidopsis* transcription factor family members were primarily derived from the Plant Transcription Factor Database, available at https://planttfdb.gao-lab.org/index.php (accessed on 14 May 2024). Following this, the COUNTIF function in Excel 2010 was utilized to identify various transcription factor family members among the 678 DEGs.

### 4.3. Quantitative Real-Time PCR

Total RNA was extracted from each sample using the RNA Easy Fast Plant Tissue Kit (Tiangen Co., Ltd., Nanjing, China). CDNA was synthesized with the lnRcute lncRNA First-Strand cDNA Kit (Tiangen Co., Ltd., Nanjing, China). Gene-specific primers for qRT-PCR were designed using Primer Premier 5.0 software; the primer list is in Supplementary Table S5. qRT-PCR was conducted using PowerUp^TM^ SYBR^TM^ Green Master Mix under the following conditions: 95 °C for 3 min, 40 cycles of 94 °C for 15 s, 60 °C for 15 s, and 72 °C for 32 s. The relative expression levels were quantified using the 2^−ΔΔCt^ method [55].

### 4.4. GO Enrichment and KEGG Pathway Enrichment Analyses of Differentially Expressed Genes

The R 4.4.0 software package Goseq was employed for Gene Ontology (GO) analysis to identify the biological functions of DEGs [56]. A GO term was considered significantly enriched if the adjusted *p*-value was < 0.05 [57]. Additionally, KEGG pathway enrichment was conducted using KOBAS 3.0 software [58] to elucidate the main biological processes in cells, including metabolism, membrane transport, and signal transduction pathways, as well as information on chemical complexes and enzyme reactions within cells. The results of the KEGG enrichment analysis (adjusted *p*-value < 0.5) were visualized using RStudio. A pathway is considered enriched if the adjusted *p*-value < 0.05.

### 4.5. Protein–Protein Interaction Analysis

A protein–protein interaction network was constructed using the STRING database (https://cn.string-db.org/ (accessed on 30 May 2024)) and visualized using Cytoscape 3.9.1 software [59].

## 5. Conclusions

The formation of stamens in plants is a highly intricate process, regulated by complex interactions within regulatory networks. In this study, we selected flowers from both wild-type and transgenic *Arabidopsis* plants for transcriptome sequencing based on the overexpression of the male-specific gene MSL-lncRNAs. We identified 678 DEGs in flowers of wild-type and transgenic plants and screened for three key genes associated with stamen development. Additionally, we identified transcription factor family members that may play a role in this process. GO enrichment analysis revealed that the DEGs were involved in processes such as pollen formation, polysaccharide catabolism, and secondary metabolism. Furthermore, KEGG pathway analysis suggested that MSL-lncRNAs may promote stamen development by upregulating genes associated with the phenylpropanoid biosynthesis pathway. Notably, genes related to tapetum formation and anther development were found to be regulated, indicating that MSL-lncRNAs might influence pollen development by positively regulating these genes. We hypothesized that within the framework of the ABC model, MSL-lncRNAs interacts with F-box-containing genes (AT5G52940, AT5G54450, and AT4G25930) in class B genes to regulate stamen development. Additionally, other transcription-factor-related genes may also be involved. MSL-lncRNAs may further regulate genes associated with tapetum formation and anther development by participating in the phenylpropanoid biosynthesis pathway, ultimately influencing pollen development. These findings provide a theoretical basis for further investigations into the genetic regulation of MSL-lncRNAs in stamen and pollen development.

## Figures and Tables

**Table 1:** Comparative statistics of sample sequencing data and the reference genome.

	Ara_WT1	Ara_WT2	Ara_WT3	Ara_EX25	Ara_EX38	Ara_EX47
Clean reads	44,288,032	38,908,522	45,654,690	40,657,346	41,144,606	40,870,856
Unmapped reads	1,060,318	940,831	1,086,060	864,511	948,668	1,305,017
Map reads	43,227,714	37,967,691	44,568,630	39,792,835	40,195,938	39,565,839
Map rate	0.9761	0.9758	0.9762	0.9787	0.9769	0.9681
Unique map reads	42,286,496	36,862,000	43,615,891	38,902,184	39,064,666	38,093,079
Unique map rate	0.9548	0.9474	0.9553	0.9568	0.9494	0.932

## Data Availability

Data are contained within the article and Supplementary Materials.

## Supplementary Materials

The following supporting information can be downloaded at: https://www.mdpi.com/article/10.3390/plants13202906/s1, Figure S1. Directed acyclic graph of biological process enrichment analysis of DEGs. Arrows signify hierarchical relationships between upper and lower layers; ellipses denote GO terms not ranked in the top 10 in terms of enrichment; boxes surround those ranked in the top 10. Colors reflect the enrichment of DEGs in GO terms, with deeper colors indicating stronger enrichment. Red represents the most pronounced enrichment, followed by yellow, while colorless indicates negligible enrichment. The top row in the box indicates the GO term number, the second row describes the term’s function, the third row shows the *p*-value, and the final row details the number of DEGs enriched in the term in the study, relative to the total number of DEGs in the term. Figure S2. Directed acyclic graph of cellular component enrichment analysis of DEGs. Arrows signify hierarchical relationships between upper and lower layers; ellipses denote GO terms not ranked in the top 10 in terms of enrichment; boxes surround those ranked in the top 10. Colors reflect the enrichment of DEGs in GO terms, with deeper colors indicating stronger enrichment. Red represents the most pronounced enrichment, followed by yellow, while colorless indicates negligible enrichment. The top row in the box indicates the GO term number, the second row describes the term’s function, the third row shows the *p*-value, and the final row details the number of DEGs enriched in the term in the study, relative to the total number of DEGs in the term. Figure S3. Directed acyclic graph of molecular function enrichment analysis of DEGs. Arrows signify hierarchical relationships between upper and lower layers; ellipses denote GO terms not ranked in the top 10 in terms of enrichment; boxes surround those ranked in the top 10. Colors reflect the enrichment of DEGs in GO terms, with deeper colors indicating stronger enrichment. Red represents the most pronounced enrichment, followed by yellow, while colorless indicates negligible enrichment. The top row in the box indicates the GO term number, the second row describes the term’s function, the third row shows the *p*-value, and the final row details the number of DEGs enriched in the term in the study, relative to the total number of DEGs in the term. Table S1. The differentially expressed genes. Table S2. The FPKM of the three with highest and the three with lowest fold change values. Table S3. GO enrichment analysis of DEGs. Table S4. KEGG enrichment analysis of DEGs. Table S5. The primer list of qRT-PCR. Table S6. The corresponding information for the IDs, names, and functional annotations. The RNA-seq data for the *Arabidopsis* samples are available at https://www.ncbi.nlm.nih.gov/bioproject/PRJNA1153351 (accessed on 14 May 2024).
