# Supplementary material for: Transcriptome Remodeling in Arabidopsis: A Response to Heterologous Poplar MSL-lncRNAs Overexpression
Source: Plants (Basel). 2024 Oct 17;13(20):2906. doi: 10.3390/plants13202906 (PMC11511487; doi:10.3390/plants13202906)
Supplement: Supplementary file 1 [file plants-13-02906-s001.zip › Supplementary Figure S2.pdf]

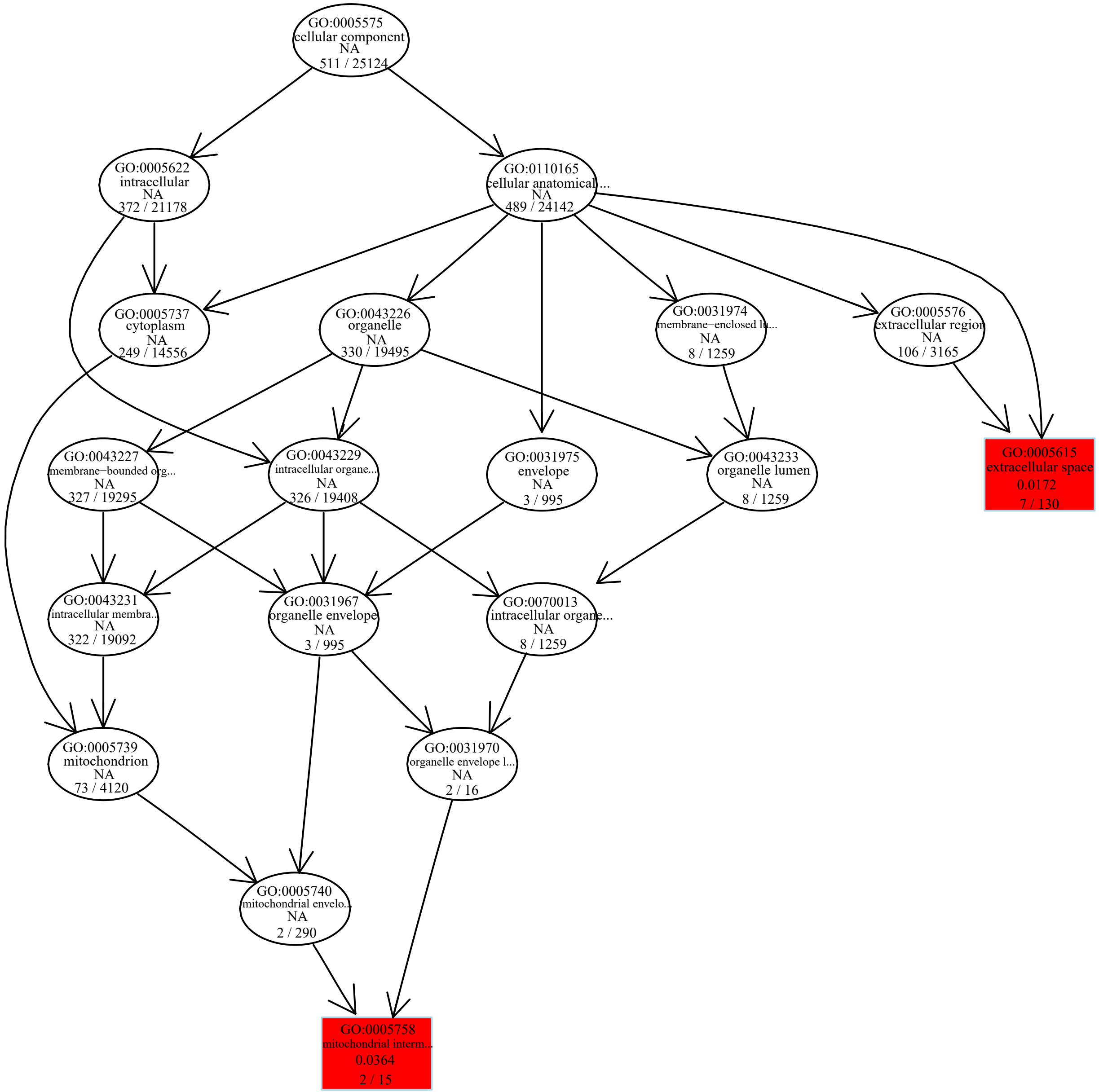

Figure S2. Directed acyclic graph of cellular component enrichment analysis of DEGs. Arrows signify hierarchical relationships between upper and lower layers; ellipses denote GO terms not ranked in the top 10 in terms of enrich-ment; boxes surround those ranked in the top 10. Colors reflect the enrichment of DEGs in GO terms, with deeper colors indicating stronger en-richment. Red represents the most pronounced enrichment, followed by yellow, while colorless indicates negligible enrichment. The top row in the box indicates the GO term number, the sec-ond row describes the term's function, the third row shows the p-value, and the final row details the number of DEGs enriched in the term in the study, relative to the total number of DEGs in the term.
