# Supplementary material for: Transcriptome Remodeling in Arabidopsis: A Response to Heterologous Poplar MSL-lncRNAs Overexpression
Source: Plants (Basel). 2024 Oct 17;13(20):2906. doi: 10.3390/plants13202906 (PMC11511487; doi:10.3390/plants13202906)
Supplement: Supplementary file 1 [file plants-13-02906-s001.zip › Supplementary Table S2.pdf]

Table S2. The FPKM of the three with highest and the three with lowest fold change values.

Gene id	Ara_WT1	Ara_WT2	Ara_WT3	Ara_EX25	Ara_EX38	Ara_EX47	logFC	P-Value
AT5G52940	0.195153	0	0.031648	0.248918	0.035142	0.145018	6.456810	0.017828
AT5G54450	0.044073	0.050573	0.042884	0.144552	0	0.049124	5.939712	0.019778
AT4G25930	0.044192	0.050711	0.043000	0	0.143240	0	5.875918	0.027108
AT1G75945	14.39309	31.57484	15.44651	0	0	0	-8.405495	1.74E-64
AT3G06835	0.202862	0.349176	0.296085	0.110892	0	0	-3.356998	0.000299
AT5G12380	0.145492	0.111301	0.141567	0	0	0	-3.352904	0.000296
