# Supplementary material for: Transcriptome Remodeling in Arabidopsis: A Response to Heterologous Poplar MSL-lncRNAs Overexpression
Source: Plants (Basel). 2024 Oct 17;13(20):2906. doi: 10.3390/plants13202906 (PMC11511487; doi:10.3390/plants13202906)
Supplement: Supplementary file 1 [file plants-13-02906-s001.zip › Supplementary Table S5.pdf]

Table S5. The primer list of qRT-PCR.

Gene ID	Forward primer sequence	Reverse primer sequence	Application
AT5G52940	AATGGAGTTGGTGGATGG	GAGGCAGAGTCACAAGAGG	qPCR
AT5G54450	AACGCTAATGAAGATGACTC	TGATGATTTGGGTTTGAC	qPCR
AT4G25930	AGAGGTGATGACCGTTGG	AGGAGGGAGCTGATGTTT	qPCR
