# Supplementary material for: Transcriptome Remodeling in Arabidopsis: A Response to Heterologous Poplar MSL-lncRNAs Overexpression
Source: Plants (Basel). 2024 Oct 17;13(20):2906. doi: 10.3390/plants13202906 (PMC11511487; doi:10.3390/plants13202906)
Supplement: Supplementary file 1 [file plants-13-02906-s001.zip › Supplementary Table S6.pdf]

Table S6. The corresponding information for the IDs, names, and functional annotations.

Gene id	Gene name	Annotation
AT5G62320	MYB99	influence the formation of pollen walls and the viability of pollen
AT5G65080	MAF5	regulates flowering time
AT2G19070	SHT	involved in pollen exine formation
AT4G20050	QRT3	involved in pollen exine formation
AT1G67990	TSM1	encodes a tapetum-specific O-methyltransferase
AT5G66690	UGT72E2	involved in lignin metabolism
AT1G74550	CYP98A9	influence the viability of pollen
AT1G62940	ACOS5	involved in pollen wall exine formation
AT1G80820	CCR2	involved in lignin biosynthesis
AT5G51890	PPX66	involved in the lignification of tracheary elements
AT5G15180	PPX56	involved in the biosynthesis and degradation of lignin
AT4G05100	MYB74	influence anther development
AT4G21440	MYB102	influence anther development
AT5G56110	MYB80	influence anther development
AT1G01280	CYP703A2	influence stamen development
AT1G02050	PKSA	influence stamen development
AT4G34850	PKSB	influence the stamen formation and development
AT4G35420	TKPR1	impairing the formation of the pollen outer wall
AT1G60300	NAC	secondary cell wall synthesis
AT1G71930	VND7	secondary cell wall synthesis
AT5G18270	NAC087	secondary cell wall synthesis
